# HDA15-Mediated Deacetylation of GPX1 Inhibits Its Nuclear Translocation and Increases Osmotic Stress Sensitivity in Rice

**DOI:** 10.3390/ijms27167073

**Published:** 2026-08-07

**Authors:** Fengchao Zhai, Xiaoyun Ma, Wenge Li, Xinyue Fan, Jing Zhang, Heng Zhou, Yanjie Xie

**Affiliations:** 1 Laboratory Center of Life Sciences, College of Life Sciences, Nanjing Agricultural University, Nanjing 210095, China; 2021216043@stu.njau.edu.cn (F.Z.); 2025116081@stu.njau.edu.cn (W.L.); 2024816148@stu.njau.edu.cn (X.F.); 2 National Key Laboratory for the Development and Utilization of Forest Food Resources, Co-Innovation Center for Sustainable Forestry in Southern China, State Key Laboratory of Tree Genetics and Breeding, Key Laboratory of State Forestry and Grassland Administration on Subtropical Forest Biodiversity Conservation, College of Life Sciences, Nanjing Forestry University, Nanjing 210037, China; 2022216007@stu.njau.edu.cn (X.M.); jingzhang@njfu.edu.cn (J.Z.)

**Keywords:** acetylation, glutathione peroxidase, histone deacetylase, osmotic stress, redox signalling, rice

## Abstract

Redox regulation plays an important role in plant stress responses. Our previous study revealed that rice GLUTATHIONE PEROXIDASE 1 (GPX1) acts as a redox sensor and transducer and promotes osmotic stress tolerance by transfer of cytosolic oxidative signals to transcription factor BASIC LEUCINE ZIPPER 68 (bZIP68). However, the mechanisms governing GPX1 activity and nuclear localization remain unclear. Here, we show that osmotic stress increases GPX1 acetylation. Peroxidase activity and subcellular localization assay indicated that the effects of acetylation on GPX1 function are site-specific, as the acetylation of K94 and K121 enhances GPX1 enzymatic activity, whereas the C-terminal K159/K162/K163 cluster is required for its nuclear translocation. Transgenic complementation and physiological assays confirmed that substitution of K159/K162/K163 sites into arginine abolished GPX1-mediated osmotic stress tolerance and the activation of bZIP68 target genes. Furthermore, we discovered that HISTONE DEACETYLASE 15 (HDA15) interacts with and deacetylates GPX1. HDA15-mediated deacetylation reduced enzymatic activity, nuclear translocation and subsequently the interaction with bZIP68 of GPX1. Accordingly, *HDA15*-overexpressing rice showed greater membrane damage, weaker induction of bZIP68-regulated genes and increased sensitivity to osmotic stress. These results identify HDA15-mediated GPX1 deacetylation as a negative regulatory mechanism that connects redox enzyme activity, protein localization and stress-responsive transcription in rice.

## 1. Introduction

Drought and soil salinity lower external water potential, restrict water uptake and reduce cell turgor, thereby limiting plant growth [1,2]. Rice (Oryza sativa) is particularly sensitive to water deficit and salinity. Therefore, understanding how it maintains cellular homeostasis under osmotic stress is important for crop improvement. Plants respond through coordinated calcium, protein kinase, abscisic acid (ABA), and transcriptional and metabolic pathways [3,4]. Osmotic stress also rapidly changes the production and removal of reactive oxygen species (ROS) [5]. Although excessive accumulation of ROS causes membrane, protein and nucleic acid damage, it can also act as a signalling messenger that contributes to stress acclimatization and metabolic changes [6,7].

Hydrogen peroxide is a major ROS signal because it can reversibly oxidize cysteine residues in proteins and play a crucial role in regulating cellular redox status [8]. Redox-based regulation is centred on the cellular thiol–redox network, which integrates reductive inputs from metabolism with oxidative ROS signals [9]. Thiol peroxidases act as sensitive peroxide and redox sensors and play a central role in this signal transduction process. Plant glutathione peroxidases are thioredoxin-dependent enzymes located in several cellular compartments, and contribute to ROS homeostasis [10,11]. In Arabidopsis, GLUTATHIONE PEROXIDASE 3 functions as both a peroxide scavenger and a redox signal transducer during ABA and drought responses [12]. A previous study also revealed that rice GPX1 serves as a redox sensor and transducer under osmotic stress [13]. Under osmotic stress, GPX1 is rapidly oxidized and forms an intramolecular disulfide bond. GPX1 then interacts with the VRE-like BASIC LEUCINE ZIPPER (bZIP) transcription factor bZIP68 and promotes its oxidation-dependent tetramerization. This increases bZIP68 binding to the promoters of osmotic stress-responsive genes, including DEHYDRATION-RESPONSIVE ELEMENT BINDING PROTEIN1 (DREB1A and DREB1B) and COLD-REGULATED413-THYLAKOID MEMBRANE1 (COR413-TM1). Osmotic stress also increases GPX1 lysine acetylation and promotes its movement from the cytoplasm to the nucleus, where it interacts with bZIP68. However, the underlying mechanism by which acetylation of GPX1 regulates the redox signal transduction during rice stress response is unclear.

Lysine acetylation is a major protein post-translational modification (PTM) that occurs at histones. Protein acetylation status is reversibly controlled by histone acetyltransferases (HACs) and histone deacetylases (HDACs). HDACs are highly conserved in eukaryotes. Large-scale studies have identified acetylated metabolic enzymes, ribosomal proteins, redox proteins and transcriptional regulators in plants [14,15,16]. Acetylation can alter protein structure, activity, stability, interactions and localization, providing a flexible mechanism for metabolic, genetic, and epigenetic regulation in response to metabolic stimuli and environmental cues [17]. Increasing evidence indicates that protein deacetylation participates in regulating plant growth and stress responses. For instance, Arabidopsis HDA6 interacts with and deacetylates BIN2, thereby suppressing its kinase activity, whereas HDA9 negatively regulates salt stress responses through interaction with WRKY53 and modulation of its DNA-binding capacity and transcriptional activity [18,19]. In rice, OsHDA702-mediated deacetylation of GSK3/SHAGGY-like kinase 2 (OsGSK2), the rice homolog of Arabidopsis BIN2, reduces OsGSK2 kinase activity and disrupts its interaction with BRASSINAZOLE-RESISTANT 1 (OsBZR1). This process facilitates OsBZR1 release and subsequently enhances the expression of brassinosteroid-responsive genes to promote lateral root formation [20]. In contrast, acetylation mediated by the rice sirtuin protein OsSRT1 suppresses the transactivation activity of glyceraldehyde-3-phosphate dehydrogenase (GAPDH), leading to reduced expression of glycolytic genes [21]. Moreover, OsHDA716 negatively regulates rice chilling tolerance by removing acetyl groups from OsbZIP46, resulting in decreased transcriptional activity and reduced protein stability of OsbZIP46 [22].

Here, we identify functionally distinct acetylation sites in GPX1. K94 and K121 are required for the acetylation-dependent increase in enzymatic activity, whereas the C-terminal K159/K162/K163 cluster is required for osmotic stress-induced nuclear accumulation. We further identify HDA15 as a GPX1-interacting deacetylase that reduces GPX1 activity, nuclear localization and association with bZIP68. HDA15 overexpression weakens activation of the GPX1-bZIP68 pathway and increases rice sensitivity to osmotic stress. These findings show that site-specific non-histone acetylation links redox enzyme activity, protein localization and stress-responsive transcription in rice.

## 2. Results

### 2.1. Acetylation Regulates Activity and Nuclear Translocation of GPX1

**Figure 1:**
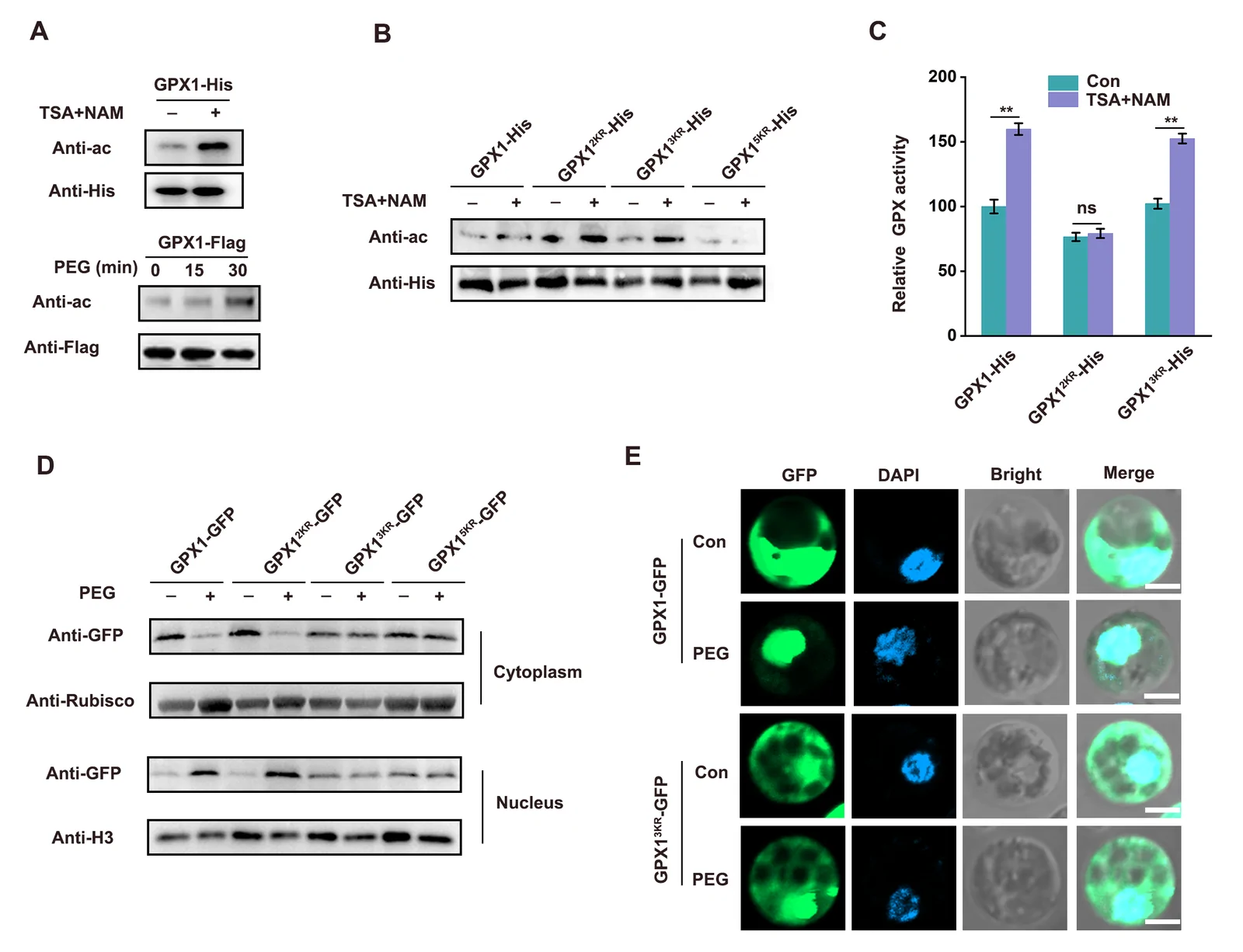
Acetylation mediates enzymatic activity and nuclear translocation of GPX1. (**A**) GPX1 is acetylated both in vitro and in vivo. Left: GPX1-His recombinant proteins were harvest from the *E. coli* treated with or without 5 μM TSA and 5 mM NAM treatment and the acetylation level were detected. Right: total protein was extracted from *GPX1*-OE1 rice plant treated with 10% PEG 6000 for indicated time and analyzed in reducing and non-reducing condition. For acetylation level determination, GPX1-Flag proteins were immunoprecipitated and analyzed by immunoblotting with anti-LysAc and anti-Flag antibodies. (**B**) Amino acid substitutions reduce GPX1 acetylation levels. GPX1-His or its mutated version harvested from the *E. coli* treated with or without 5 μM TSA and 5 mM NAM treatment were detected by immunoblotting with the anti-LysAc antibody. GPX1^2KR^ indicated K94 and K121 were substituted into arginine, GPX1^3KR^ indicated K159, K162, and K163 were substituted into arginine, GPX1^5KR^ indicated all five lysines were substituted into arginine. (**C**) Acetylation enhances GPX1 enzymatic activity. GPX1-His, GPX1^2KR^-His, and GPX1^3KR^-His recombinant protein harvest from the *E. coli* treated with or without 5 μM TSA and 5 mM NAM treatment were used for GPX activity assay. Data are presented as mean ± SD (*n* = 3). ** indicates significant difference compared with control (*p* < 0.01), ns indicates no significant (Student *t*-test). (**D**) Acetylation promotes GPX1 nuclear localization. Cytoplasmic and nuclear fractions from rice protoplasts expressing GPX1 or its acetylation-site variants, with or without 10% PEG 6000 treatment for 30 min, were analyzed by immunoblotting with an anti-GFP antibody. Rubisco and histone H3 were used as markers for cytoplasmic and nuclear fractions, respectively. GPX1-Flag proteins isolated from nuclear extracts were immunoprecipitated and examined using anti-acetyl-lysine and anti-Flag antibodies. (**E**) Mutation of K159, K162, and K163 compromises stress-induced GPX1 nuclear accumulation. Rice protoplasts expressing pAN580-GPX1-GFP or pAN580-GPX1^3KR^-GFP were treated with or without 10% PEG 6000 for 30 min. GFP fluorescence was monitored by confocal microscopy, and nuclei were visualized by DAPI staining. Images represent four independent experiments. Scale bars, 10 μm.

To confirm whether GPX1 undergoes acetylation, we detect the acetylation level of GPX1 both in vitro and in vivo. The immunoblot results clearly showed that histone deacetylase inhibitors including trichostatin A (TSA) and nicotinamide (NAM) and PEG treatment significantly enhance the acetylation levels of GPX1 (Figure 1A). To identify acetylation sites in GPX1, purified GPX1-His recombinant protein from *E. coli* with TSA and NAM treatment was analyzed by mass spectrometry. Four acetylation sites (K94, K121, K159, K162,) in GPX1 were identified (Figure S1).

Posttranslational modification like acetylation/deacetylation usually affects protein function or subcellular localization. Thus, we first determine whether acetylation affects the activity of GPX1. The results showed that TSA and NAM treatment significantly enhances the activity of GPX1-His recombinant protein (Figure S2). To examine whether this acetylation affects the GPX1 activity, these acetylated lysine residues of GPX1 were substituted with arginine (R) to mimic deacetylated lysine. First, we generated GPX1^K94R^, GPX1^K121R^, GPX1^K159R^, and GPX1^K162R^ recombinant protein. The GPX activity results showed that mutation in K94 and K121 significantly attenuated TSA and NAM treatment-induced increase in GPX1 activity (Figure S2). As the K163 were closed to K159, K162 and located in the C terminal of the GPX1 protein, we then further generated GPX1^2KR^ (GPX1^K94,121R^), GPX1^3KR^ (GPX1^K159,162,163R^) and GPX1^5KR^ (GPX1^K94,121,159,162,163R^) recombinant proteins. The immunoblot results showed that only mutation in all five lysine residues could abolish TSA and NAM treatment-induced increases in acetylation level (Figure 1B), indicating that K94, K121, K159, K162, and K163 are major acetylation sites. Further activity results confirmed that K94 and K121 are responsible for the acetylation-induced increase in GPX1 activity (Figure 1C and Figure S2).

As our previous study indicated that acetylation affects the nuclear translocation of GPX1 [13], we then determine the major acetylation sites responsible for GPX1 nuclear translocation. The rice protoplasts were transfected with GPX1-GFP, GPX1^2KR^-GFP, GPX1^3KR^-GFP, and GPX1^5KR^-GFP and then cytoplasmic and nuclear proteins were isolated. Immunoblot results showed that higher levels in the nuclear fraction and lower levels in cytoplasmic part of GPX1-GFP and GPX1^2KR^-GFP proteins were observed after PEG treatment than under control condition (Figure 1D). However, the protein levels of GPX1^3KR^-GFP and GPX1^5KR^-GFP in both the cytoplasm and nucleus exhibited no difference between control condition and PEG treatment (Figure 1D). This result indicated that K159/K162/K163 are the key sites for stress-induced GPX1 nuclear accumulation. To confirm this, the subcellular localization was further detected by confocal imaging of rice protoplasts. Consistently, the results showed that the GPX1-GFP protein, but not GPX1^3KR^-GFP, accumulated in the nucleus upon PEG treatment (Figure 1E). Together, these results clearly suggested that acetylation of K159, K162, and K163 is responsible for stress-induced nuclear translocation of GPX1.

### 2.2. Acetylation Is Required for GPX1 Conferred Rice Osmotic Stress Tolerance

To determine the physiological relevance of GPX1 acetylation, we generated complementation lines expressing either wild-type GPX1 or acetylation-deficient GPX1 variants under the control of the endogenous *GPX1* promoter in the *gpx1* mutant background (*GPX1pro:GPX1 gpx1*, *GPX1pro:GPX1^2KR^* gpx1, *GPX1pro:GPX1^3KR^ gpx1*, and *GPX1pro:GPX1^5K^*^R^ *gpx1*). Immunoblot analysis confirmed comparable accumulation of GPX1 proteins among the generated transgenic lines, allowing further phenotypic evaluation (Figure S3).

**Figure 2:**
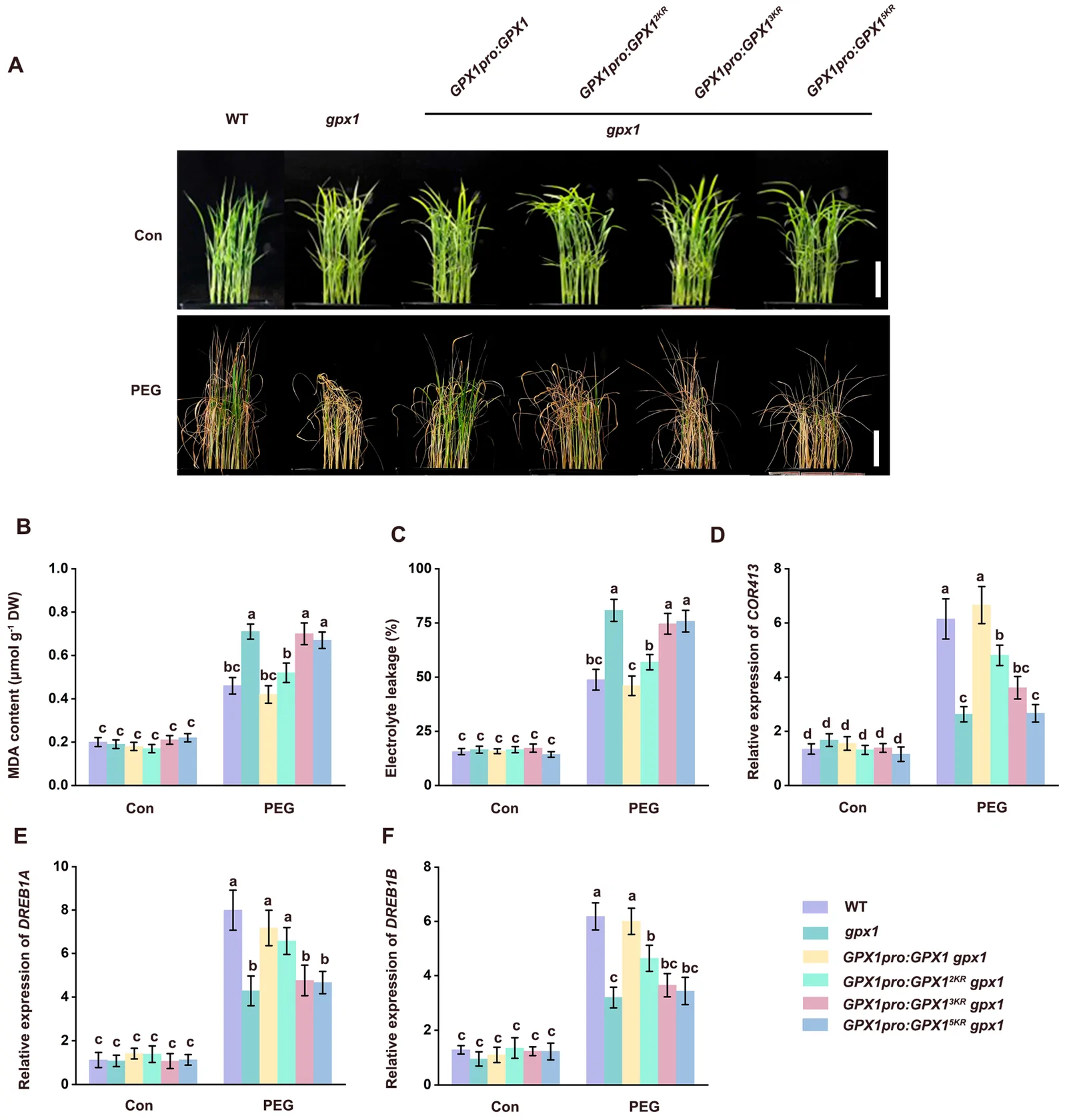
Acetylation is essential for GPX1-mediated rice osmotic stress tolerance. (**A**) Photographs of wild type, *gpx1*, *GPX1pro:GPX1 gpx1*, *GPX1pro:GPX1^2KR^ gpx1*, *GPX1pro:GPX1^3KR^ gpx1*, and *GPX1pro:GPX1^5KR^ gpx1* exposed to 20% PEG 6000 for 10 d. Thirty plants per line were used per replicate. Scale bars, 10 cm. (**B**) Malondialdehyde (MDA) content of leaves from rice seedlings exposed to PEG treatment for 4 d. (**C**) Electrolyte leakage of rice seedlings exposed to 20% PEG 6000 for 4 d. (**D**–**F**) Expression of bZIP68 target genes (*COR413-TM1*, *DREB1A*, and *DREB1B*) in wild type, gpx1, and different GPX1 complementary lines upon PEG treatment. Two-week-old rice seedlings were treated with 20% PEG 6000 for 2 d, and leaves were harvested for RT-qPCR analysis. Related gene expression was normalized to *OsActin1* and *OsActin2*. In (**B**–**F**), Data are presented as mean ± SD (*n* = 3). Different letters denote significant differences at *p* < 0.05 (one-way ANOVA, Duncan’s multiple range tests). All experiments were repeated at least three times with similar results.

Under normal growth conditions, all complemented plants displayed similar developmental performance. However, following PEG-induced osmotic stress, the *gpx1* mutant exhibited pronounced growth inhibition compared with wild-type plants (Figure 2A). Expression of wild-type GPX1 effectively restored osmotic stress tolerance in the mutant background, whereas complementation with GPX1^2KR^ resulted in only partial recovery. In contrast, plants expressing GPX1^3KR^ or GPX1^5KR^ showed limited improvement, indicating that acetylation-deficient GPX1 variants were unable to fully compensate for the loss of endogenous GPX1 function. Consistent with these observations, *gpx1* mutants exposed to PEG treatment accumulated higher levels of malondialdehyde (MDA) and exhibited increased electrolyte leakage, reflecting enhanced membrane damage and lipid peroxidation (Figure 2B,C). These physiological defects were efficiently alleviated by wild-type GPX1 but were only partially recovered by GPX1^2KR^, and minimally recovered by GPX1^3KR^ or GPX1^5KR^.

To further explore how GPX1 acetylation contributes to osmotic stress responses, we analyzed the expression of known bZIP68 target genes, including *COR413-TM1*, *DREB1A*, and *DREB1B*. PEG-induced activation of these downstream genes was strongly promoted by wild-type GPX1 but was reduced in plants expressing acetylation-site mutants, especially GPX1^3KR^ and GPX1^5KR^ (Figure 2D–F). Collectively, these results demonstrate that acetylation of K159, K162, and K163 is essential for GPX1-mediated osmotic stress tolerance in rice.

### 2.3. GPX1 Interacts with Histone Deacetylase HDA15

**Figure 3:**
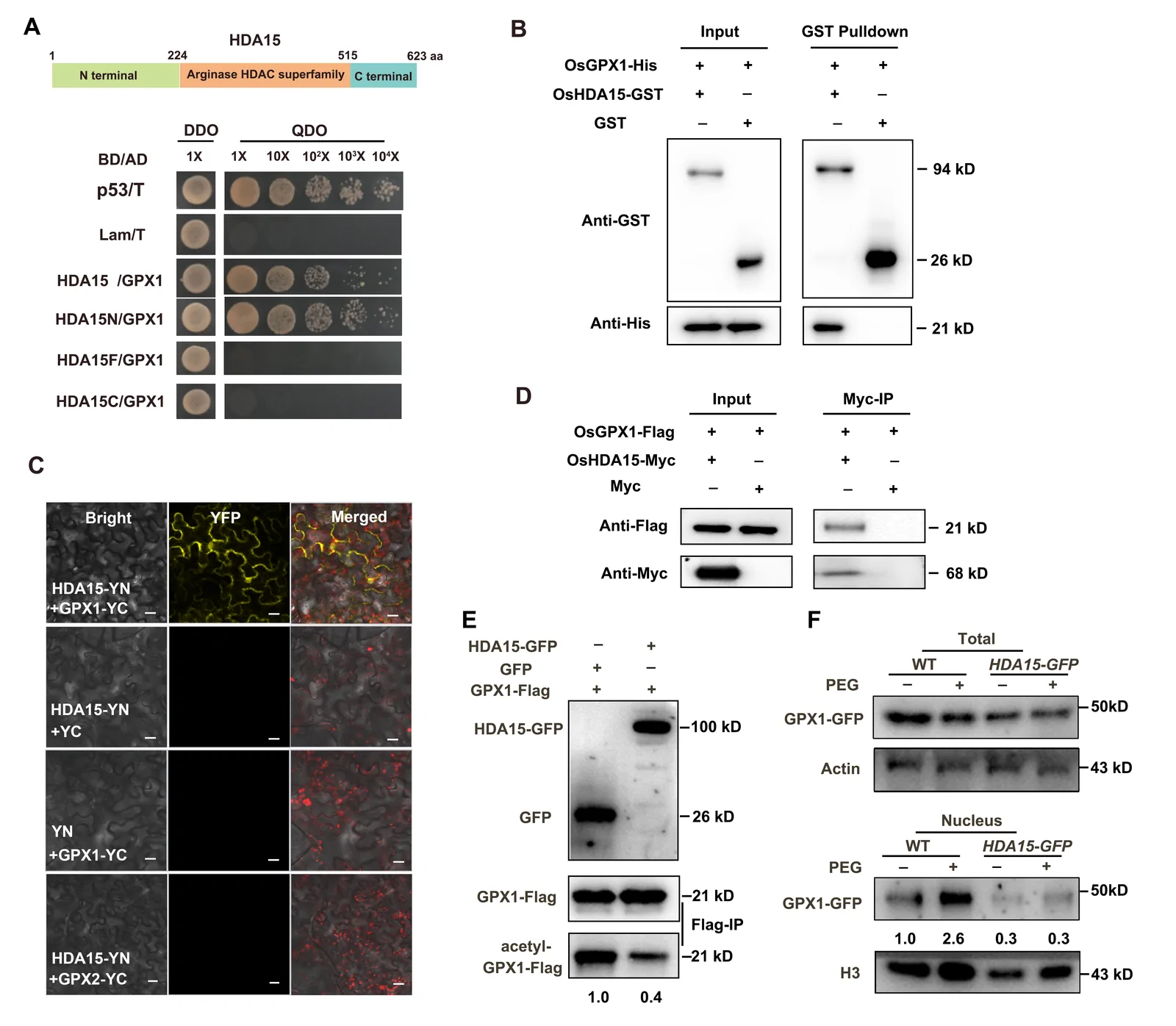
HDA15 interacts with and deacetylates GPX1. (**A**) Identification of the HDA15 region responsible for interaction with GPX1. Full-length HDA15 and three truncated fragments were tested for interaction with GPX1 using yeast two-hybrid assays. p53 and Lamin were used as positive and negative controls, respectively. (**B**) Verification of the GPX1–HDA15 interaction using GST pull-down assays. Purified GST-HDA15 and His-GPX1 proteins were incubated together, and bound proteins were detected by immunoblot analysis. (**C**) Verification of the interaction between GPX1 and HDA15 in plant cells by uisng BiFC assay. GPX1-YC and HDA15-YN fusion proteins were transiently expressed in *Nicotiana benthamiana* leaves, and protein interaction was examined by confocal imaging. GPX2-YC and HDA15-YN were used as negative control. Scale bars, 20 μm. (**D**) Co-immunoprecipitation analysis confirms the association between GPX1 and HDA15. HDA15-Myc or empty vector controls were transiently introduced into *GPX1*-OE1 rice protoplasts. Protein complexes were immunoprecipitated using anti-Myc antibodies and detected with anti-Flag and anti-Myc antibodies. Input samples were analyzed before immunoprecipitation. (**E**) HDA15 deacetylates GPX1 in vivo. HDA15-GFP or GFP control constructs were expressed in *GPX1*-OE1 rice protoplasts. GPX1-Flag was immunoprecipitated with anti-Flag antibodies, and acetylation levels were examined using anti-acetyl-lysine antibodies. (**F**) HDA15 suppresses stress-induced GPX1 nuclear accumulation. GPX1-Flag was expressed in protoplasts derived from wild-type or *35S:HDA15-GFP* rice plants. Total and nuclear proteins from control and PEG-treated samples were analyzed by immunoblotting using anti-GFP antibodies. Actin and histone H3 served as loading controls. Relative nuclear GPX1-GFP abundance was normalized to wild-type control samples.

To unravel the enzyme(s) responsible for GPX1 acetylation, we screened known histone deacetylases for interaction with GPX1 by Y2H assay (Figure S4). We thus identified HISTONE DEACETYLASE15 (HDA15) as a GPX1-interacting protein in yeast cells. To dissect the region of HDA15 which is responsible for its interaction with GPX1, HDA15 proteins were divided into three parts (Figure 3A). Y2H assay showed that the N-terminal domain of HDA15 (amino acids 1 to 224) mediated its interaction with GPX1 (Figure 3A).

We then sought to validate the interaction between GPX1 and HDA15 next by GST pull-down, BiFC, and Co-IP assays. First, purified GPX1-His recombinant protein was incubated with a recombinant glutathione S-transferase (GST)-HDA15 fusion protein, and the pull-down experiment was performed with glutathione beads. The results showed that GPX1-His was efficiently pulled down by GST-HDA15 (Figure 3B). Then the interaction of HDA15 and GPX1 was further verified by the BiFC assay. We transiently co-infiltrated *N. benthamiana* leaves with HDA15-YN and GPX1-YC and observed the reconstitution of YFP signal (Figure 3C). Moreover, we also performed Co-IP assays in rice protoplasts to confirm the in vivo interaction between GPX1 and HDA15, in which HDA15-Myc was used with immunoprecipitated GPX1-Flag, which were detected by anti-Flag antibody. Immunoblot analysis showed that GPX1-Flag was co-immunoprecipitated by HDA15-Myc, but not Myc alone (Figure 3D). Taken together, our data suggest that HDA15 specifically interacts with GPX1 both in vitro and in planta.

To explore whether HDA15 regulates the acetylation levels of GPX1, we purified recombinant GPX1-His protein harvested from *E. coli* treated with 5 μM TSA and 5 mM NAM treatment and performed the in vitro deacetylation assay with HDA15-GST protein. Immunoblot results indicated that HDA15 deacetylates GPX1 in vitro (Figure S5A). To further investigate whether HDA15 deacetylates GPX1 in plants, we transiently transfected protoplasts prepared from *35S:GPX1-Flag* transgenic plant with *GFP* or *HDA15-GFP*. Compared with *GFP*, the transient expression of *HDA15-GFP* significantly decreased the acetylation level of GPX1, indicating that HDA15 can deacetylate GPX1 in planta (Figure 3E).

### 2.4. HDA15-Mediated Deacetylation Inhibits GPX1 Activity and Nuclear Translocation

As acetylation increases GPX1 activity, we then determine whether HDA15 affects GPX1 activity. Recombinant GPX1-His proteins harvested from *E. coli* treated with 5 μM TSA and 5 mM NAM treatment were used for in vitro deacetylation assay by GST or HDA15-GST recombinant protein. The results indicated that HDA15 significantly decreases GPX1 activity (Figure S5B).

To evaluate the importance of HDA15 in the regulation of GPX1 cyto-nuclear shuttling as a function of acetylation, we investigated the changes in localization experienced by GPX1 in protoplasts prepared from wild-type and *35S:HDA15-GFP* transgenic plants. Immunoblot analysis revealed that GPX1 accumulates to lower levels in the nucleus of *35S:HDA15-GFP* protoplasts than in that of wild-type protoplasts under normal conditions (Figure 3F). In addition, the PEG-induced nuclear accumulation of GPX1 was significantly blocked in *35S:HDA15-GFP* protoplasts relative to wild type. These results suggested that HDA15-mediated deacetylation negatively regulates GPX1 activity and nuclear localization.

### 2.5. HDA15-Mediated Deacetylation Inhibits the Interaction Between GPX1 and bZIP68 in Nucleus

**Figure 4:**
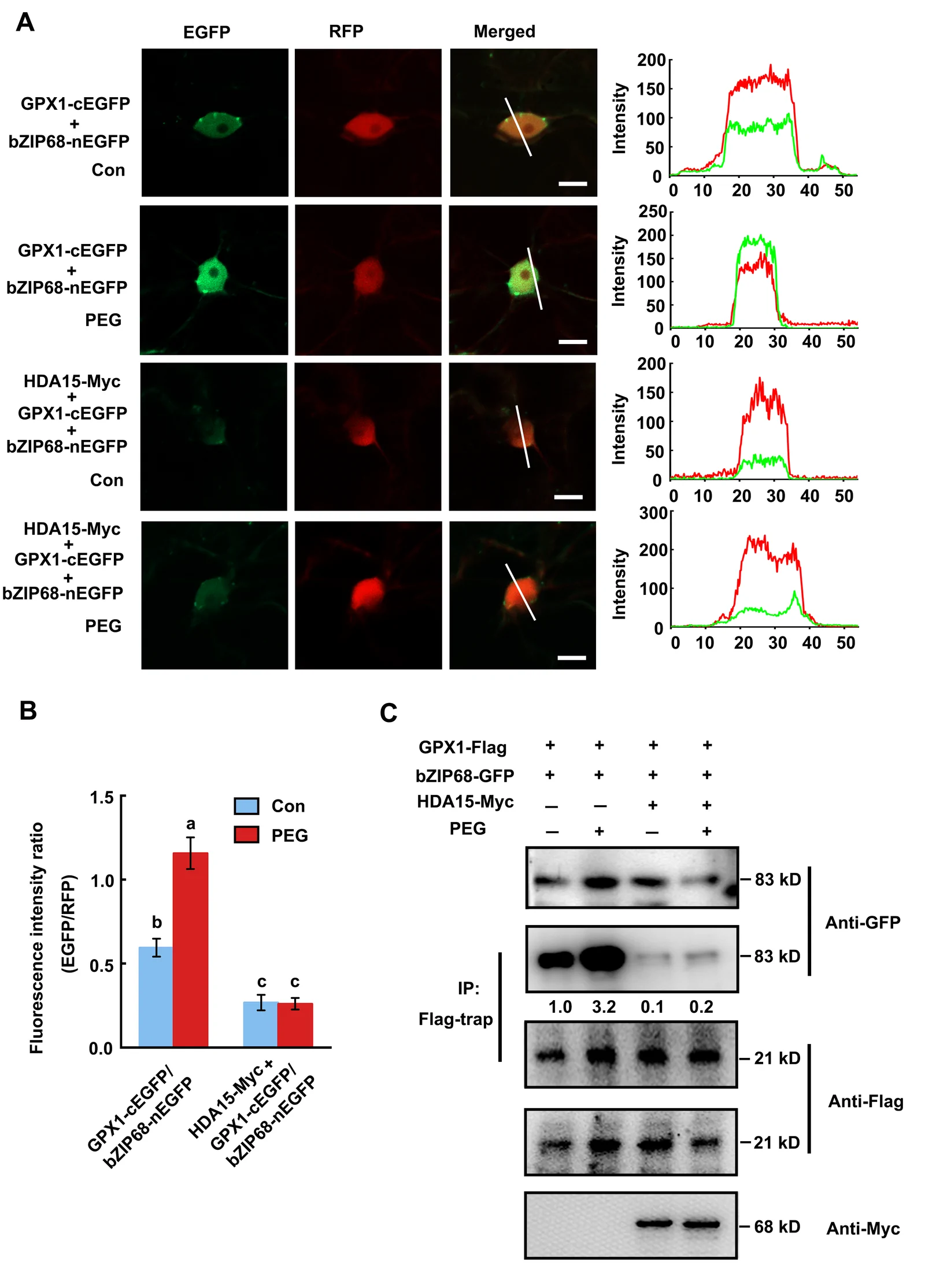
HDA15 prevents the interaction between GPX1 and bZIP68 in nucleus. (**A**) rBiFC confocal images showing that HDA15 inhibits the interaction between GPX1 and bZIP68 in plants. The fluorescent signals for GFP (BiFC, green line) and RFP (reference, red line) were determined along a line drawn on the confocal images using ImageJ2 software. Data are mean ± SD (n= 10 images). Scale bar, 10 μm. Different letters denote significant differences at *p* < 0.05 (one-way ANOVA, Duncan’s multiple range tests). (**B**) Relative fluorescence intensity ratio in (**A**). (**C**) Co-immunoprecipitation (Co-IP) assays showing that HDA15 inhibits the interaction between GPX1 and bZIP68 in plants. *N. benthamiana* leaves transiently transfected with *GPX1-Flag*, *bZIP68-GFP*, and *HDA15-Myc* or *Myc* were treated with 20% PEG 6000 for 30 min and then immunoprecipitated using Flag-Trap agarose beads. Immunoprecipitated proteins were probed using anti-Myc and anti-GFP antibodies. The quantification of the immunoblot results was normalized to GPX1-Flag after Flag-Trap and the relative protein levels of bZIP68-GFP in the control condition and without co-expression of HDA15 were defined as 1. All experiments were repeated at least three times with similar results.

To evaluate the consequences of HDA15-mediated deacetylation of GPX1 in the context of the GPX1-bZIP68 interaction, we applied a newly developed ratiometric bimolecular fluorescence complementation (rBiFC) assay [23]. For this system, we cloned *bZIP68* and *GPX1* simultaneously into a single vector backbone and in frame with the coding sequences for the N or C terminus of enhanced green fluorescence protein (eGFP), respectively. A monomeric red fluorescent protein (*RFP*) gene driven by the 35S promoter was included in the vector as an internal control. Confocal microscopy demonstrated the interaction of GPX1 with bZIP68 in the nucleus (Figure 4A,B). However, overexpression of *HDA15* significantly weakened the interaction between GPX1 and bZIP68. Moreover, the interaction between GPX1 and bZIP68 in the nucleus was strongly enhanced upon PEG treatment, but almost abolished when *HDA15* was co-transfected (Figure 4A,B). To confirm this result, we performed immunoblot analysis on protein extracts from *N. benthamiana* leaves transiently infiltrated with *GPX1-Flag*, *bZIP68-GFP* and/or *HDA15-Myc* and immunoprecipitated with anti-Flag antibody. We observed a strong interaction between GPX1 and bZIP68 that was enhanced by PEG treatment; however, the addition of HDA15 significantly blocked the interaction between GPX1 and bZIP68 both under normal conditions and upon PEG treatment (Figure 4C), which is consistent with the rBiFC results (Figure 4A). Together, these results demonstrate that HDA15-mediated deacetylation prevents the interaction of GPX1 and bZIP68.

### 2.6. HDA15 Negatively Regulates Rice Osmotic Stress Tolerance

**Figure 5:**
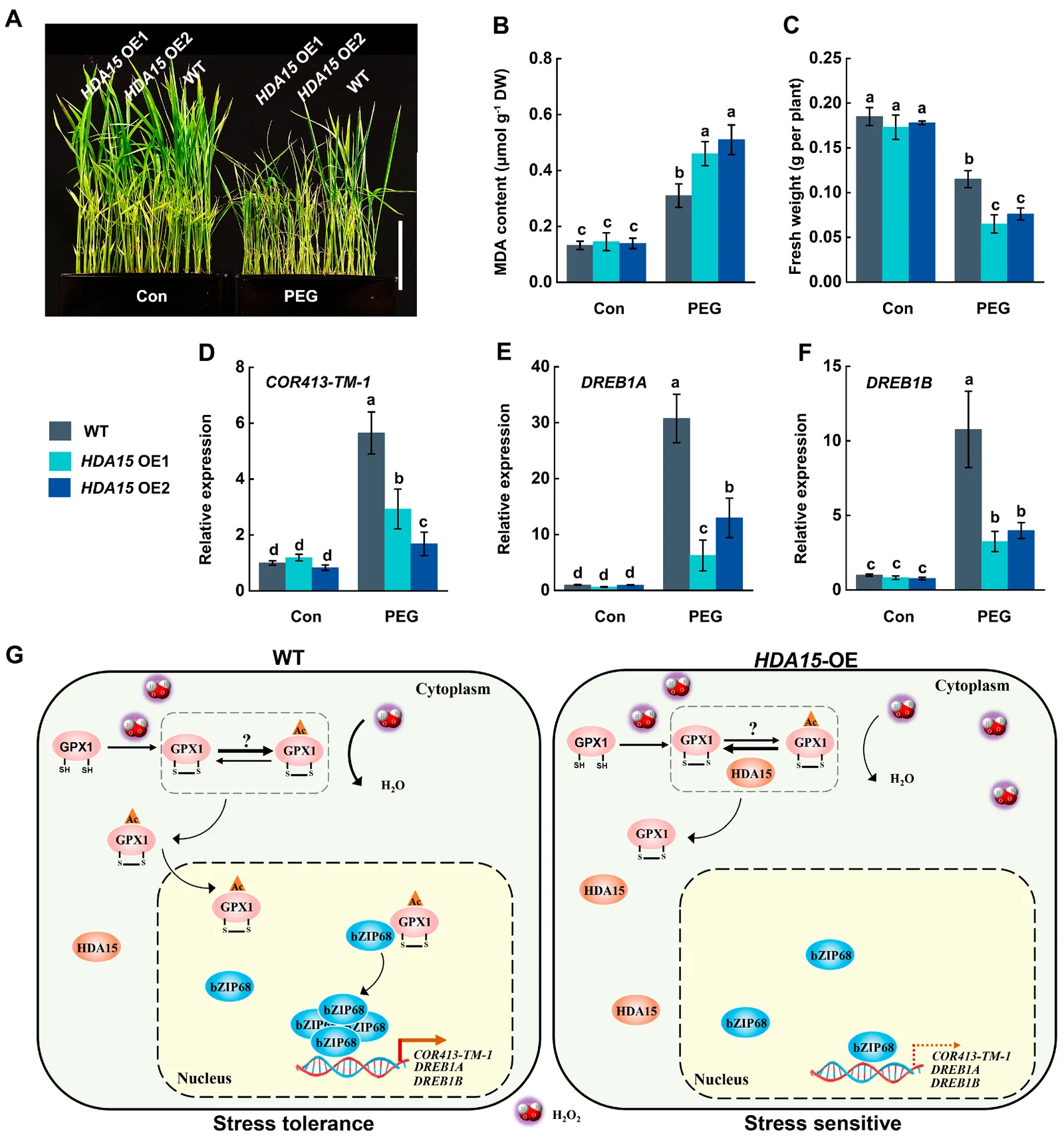
Overexpression of *HDA15* enhance rice osmotic stress sensitivity. (**A**) Photographs of wild type and the *HDA15* overexpression lines exposed to 20% PEG 6000 for 10 d. Thirty plants per line were used per replicate. Scale bars, 10 cm. (**B**) Malondialdehyde (MDA) content of leaves from rice seedlings exposed to PEG treatment for 4 d. (**C**) Fresh weight of rice seedlings exposed to 20% PEG 6000 for 10 d (**D**–**F**) Expression of bZIP68 target genes (*COR413-TM-1*, *DREB1A*, and *DREB1B*) in wild type and the *HDA15* overexpression lines upon PEG treatment. Two-week-old rice seedlings were treated with 20% PEG 6000 for 2 d, and leaves were harvested for RT-qPCR analysis. Related gene expression was normalized to *OsActin1* and *OsActin2*. (**G**) A model illustrating HDA15-mediated deacetylation inhibits GPX1 activity and nuclear translocation, thus enhancing rice osmotic stress sensitivity. In wild type, when cells perceive osmotic stress, the ensuing accumulation of H_2_O_2_ leads to GPX1 oxidation and the formation of an intramolecular disulfide bond. Subsequently, osmotic stress-induced acetylation enhances GPX1 activity and triggers its translocation from cytoplasm to nucleus. In nucleus, GPX1interacts with and oxidizes bZIP68, causing oligomerization that activates the expression of osmotic-responsive genes, thus conferring plant tolerance to osmotic stress in rice. In *HDA15*-OE plant, overaccumulated HDA15 interacts with and deacetylates GPX1, and thus inhibits GPX1 activity and nuclear translocation, further attenuates the activation of osmotic-responsive genes, and exhibits stress sensitivity. However, the sources of the osmotic stress-induced acetylation of GPX1 are still not clear. In (**B**–**F**), data are presented as mean ± SD (*n* = 3). Different letters denote significant differences at *p* < 0.05 (one-way ANOVA, Duncan’s multiple range tests). All experiments were repeated at least three times with similar results.

To elucidate the effects of HDA15 on rice osmotic stress tolerance, we generated two independent transgenic lines overexpressing *HDA15* under the control of the CaMV 35S promoter (*HDA15*-OE1 and *HDA15*-OE2). Osmotic tolerance of these transgenic lines was then evaluated. Results showed that *HDA15* OE-1 and *HDA15* OE-2 plants exhibited more severe wilting and chlorosis than the wild-type after treatment with PEG (Figure 5A). Moreover, we observed significantly higher levels of MDA and lower fresh weight in transgenic plants relative to wild-type plants (Figure 5B,C). Consistently, the induced expression of bZIP68-targeted genes triggered by PEG treatment, such as *COR413-TM1*, *DREB1A*, and *DREB1B*, were largely impaired in *HDA15* OE-1 and *HDA15* OE-2 plants (Figure 5D–F). Taken together, these results suggested that HDA15 negatively regulates plant osmotic stress tolerance.

## 3. Discussion

Osmotic stress caused by drought or salinity lowers external water potential, restricts water uptake and reduces cellular turgor, with subsequent effects on membrane integrity, metabolism, growth and survival. Plants respond through interconnected sensing and signalling systems that include calcium influx, mitogen-activated protein kinase cascades, transcriptional reprogramming, osmolyte accumulation and antioxidant defence [1,3,24]. ROS-mediated redox regulation is involved in fine-tuning those processes, while the biological specificity of the ROS signal depends on its production, and how it is sensed, transferred between proteins and delivered to the appropriate cellular compartment. Our previous study revealed that rice thiol peroxidases GPX1 acts as a redox sensor and transducer [13]. It can perceive cytoplasmic ROS signals and translocate into nucleus in an acetylation-dependent way and promote the oxidation of bZIP68, thus regulating osmotic-responsive gene expression. However, how the acetylation status of GPX1 was controlled, and the function of acetylation on GPX1 function in response to osmotic stress, remains unclear. In this study, we showed that HDA15 is responsible for the deacetylation of GPX1, and inhibits its activity and nuclear translocation, thereby impairing activation of bZIP68 and rice osmotic stress tolerance.

Lysine acetylation involves the transfer of an acetyl group from acetyl-CoA to the epsilon-amino group of a lysine residue. It often affects protein structure, activity, stability, or subcellular localization. Although early plant studies focused mainly on histones and chromatin, acetyl-proteomic analyses have revealed extensive acetylation of metabolic enzymes, ribosomal proteins, redox proteins and transcriptional regulators [14,15,16]. Here, we show that osmotic stress increases GPX1 acetylation at K94, K121, K159, K162 and K163 (Figure 1). K94 and K121 are required for the acetylation-dependent increase in GPX1 activity, while the acetylation of the C-terminal K159/K162/K163 cluster is required for stress-induced nuclear accumulation (Figure 1 and Figure S2). This observation suggests that acetylation has dual effects on GPX1 function. Similar site selectivity has also been observed in other plant proteins. Acetylation of OsECT3 is concentrated at several residues in its YTH domain, but K471 makes the predominant contribution to m6A recognition [17]. More broadly, distinct phosphorylation clusters in the immune regulator NPR1 have different effects on SUMOylation, promoter association and proteasome-dependent turnover, illustrating how modification position can partition protein function [25,26]. For GPX1, acetylation of K94 and K121 may change local charge, conformation or access of thioredoxin-related reducing partners, thereby altering catalytic turnover. In contrast, neutralization of the clustered C-terminal lysines may expose a cryptic import determinant, create a docking surface for an import factor or slow nuclear export as the nuclear localization signals in the protein are comprised of a short and continuous alkaline amino acids-enriched sequence [27]. Further structural analysis, site-specific incorporation of acetyl-lysine and direct measurements of nuclear import and export will be required to define how each acetylation module controls GPX1. We also observed that complementation with GPX1^3KR^ could not rescue the phenotype of *gpx1* mutant and the induction of bZIP68 target genes under PEG treatment (Figure 2). These results indicate that nuclear translocation is critical for GPX1 function, further highlighting the importance of GPX1’s role as a redox transducer.

The balance of protein acetylation is established asymmetrically. Acetyl groups can be added through both enzyme-catalyzed and non-enzymatic reactions. By contrast, removal of an epsilon-N-acetyllysine group requires a deacetylase. The recent finding that ACLA2-derived acetyl-CoA contributes to OsECT3 acetylation under cold stress further shows that substrate acetylation can be controlled by metabolism [17]. In this study, we identified that a histone deacetylase, HDA15, interacts with and deacetylates GPX1 in vitro and in plant cells (Figure 3). The N-terminal region of HDA15 was sufficient for interaction with GPX1, suggesting that substrate recognition can be separated from the conserved catalytic region. Recent studies have reported the function and regulatory mechanism of HDACs in regulating rice stress responses. For example, OsHDA716 deacetylates OsbZIP46 to reduce transcriptional activity and accelerate its ubiquitin-associated degradation, thus negatively regulating rice chilling tolerance [22]. In this study, we found that HDA15-mediated deacetylation inhibits GPX1 activity and its nuclear translocation after PEG treatment (Figure 3 and Figure S5). Further investigation on the whether HDA15-mediated deacetylation affects GPX1 protein stability is needed, because acetylation can compete with ubiquitination for the same lysine, alter exposure of nearby ubiquitination sites or change recruitment of E3 ligases. In OsbZIP46, deacetylation by OsHDA716 is accompanied by increased ubiquitination and faster proteasome-dependent degradation [22].

Many studies have shown that different PTMs may synergistically or antagonistically regulate protein function. Although we did not detect other PTMs of GPX1 in response to osmotic stress, our previous study showed that GPX1 also undergoes succinylation, and its succinylation level was decreased upon oxidative stress [28]. This result indicated that the function of GPX1 may also regulate by succinylation, as PEG treatment usually causes oxidative damage. Moreover, it has now been discovered that, besides the sirtuins, the other HDAC family has been found to catalyze the removal of the acyl groups other than acetyl, such as formyl, crotonyl, and myristoyl [29,30,31]. It indicated that HDA15 may also regulate the desuccinylation of GPX1. Thus, the functions and regulatory mechanisms of different PTMs on GPX1 in response to environmental change should be investigated further.

Our previous study showed that osmotic stress oxidizes GPX1 and promotes formation of an intramolecular disulfide bond between Cys71 and Cys90. Oxidized GPX1 interacts with bZIP68 and transfers the oxidative signal to bZIP68 Cys245, promoting bZIP68 tetramerization, and activation of *COR413-TM1*, *DREB1A* and *DREB1B* [13]. Our data suggest that this redox relay is spatially gated by acetylation. The K159/K162/K163 cluster is required for GPX1 accumulation in the nucleus, and HDA15-mediated deacetylation restricts nuclear GPX1 and weakens its interaction with bZIP68 under both control and PEG-treated conditions (Figure 4). Oxidation and acetylation can therefore be viewed as complementary requirements: cysteine oxidation gives GPX1 the chemical competence to transfer an oxidative equivalent, whereas lysine acetylation provides access to the nuclear compartment in which the relevant substrate is located. This observation supports a crosstalk between acetylation and oxidation in plant osmotic stress responses. Furthermore, our results showed HDA15-mediated deacetylation inhibits GPX1 activity (Figure 3 and Figure S5), as GPX1 functions as both a redox sensor and scavenger [13]. The interaction between GPX1 and HDA15 may also affect the affinity of GPX1 to sense H_2_O_2_.

Overall, our data indicate that acetylation and protein oxidation act synergistically to control the GPX1-bZIP68 module, thereby fine-tuning the regulation of osmotic stress responses temporally and spatially. HDA15 may act as an opposing checkpoint that limits this access and may help prevent inappropriate activation of bZIP68 when stress-derived ROS are absent or transient (Figure 5G).

Such cooperation between PTMs is a recurrent feature of plant stress signalling. In Arabidopsis, the E3 ligase HOS1 ubiquitinates ICE1 and promotes its degradation, whereas OST1-dependent phosphorylation stabilizes ICE1 in part by opposing HOS1 action, thereby sustaining CBF expression during cold stress [32,33]. A related mechanism operates in rice, where OsMAPK3-dependent phosphorylation of OsICE1/OsbHLH002 prevents ubiquitination and stabilizes the transcription factor during chilling. Acetylation–ubiquitination interplay is also evident for OsbZIP46, for which OsHDA716-mediated deacetylation facilitates ubiquitination and degradation [22]. These examples show that PTMs need not act independently: one modification can determine whether another occurs, change its consequence or direct the modified protein to a different cellular location. Overall, the study supports a model in which PTM crosstalk converts a broadly distributed ROS signal into transcriptional response.

## 4. Materials and Methods

### 4.1. Plant Materials and Growth Conditions

The rice (*Oryza sativa* L.) cultivar Wuyunjing 7 (WYJ-7) and its derived transgenic lines were used in this study. To generate *GPX1* complementation materials, the 35S promoter in the 1300221-Flag vector was replaced with a 2-kb native promoter region upstream of the *GPX1* coding sequence. The coding regions of wild-type *GPX1* and acetylation-deficient variants were subsequently inserted downstream of the endogenous *GPX1* promoter and upstream of the Flag tag. These constructs were introduced into the *gpx1* mutant background through Agrobacterium-mediated transformation. Homozygous transgenic plants were selected based on hygromycin resistance and confirmed by anti-Flag immunoblotting before further analyses (Figure S3).

For generation of *HDA15*-overexpressing rice plants, the full-length *HDA15* coding sequence was cloned into the 1305-gGFP vector provided by Professor Aying Zhang (College of Life Sciences, Nanjing Agricultural University). The resulting construct was transformed into rice via Agrobacterium-mediated transformation, and homozygous T3 generation plants were selected for subsequent experiments.

For osmotic stress treatment, rice seeds were surface sterilized with 5% sodium hypochlorite for 20 min and germinated in darkness at 28 °C. After germination, seedlings were transferred to 96-well hydroponic boxes containing half-strength Kimura B nutrient solution and cultivated under controlled growth conditions (16 h light/8 h dark photoperiod, 28/24 °C day/night temperature, 60% relative humidity, and 200 μmol m^−2^ s^−1^ light intensity). Two-week-old seedlings were subsequently transferred to half-strength Kimura B nutrient solution supplemented with or without 20% PEG 6000 and maintained for 10 d as previously described [13].

### 4.2. Measurement of Malondialdehyde Content

Malondialdehyde (MDA) was measured using the thiobarbituric acid method [34]. Fresh tissue (0.5 g) was ground in ice-cold 10% trichloroacetic acid with quartz sand. The homogenate was centrifuged at 4000× *g* for 20 min at 4 °C, and the supernatant was collected. Equal volumes of extract and 0.67% (*w*/*v*) thiobarbituric acid were mixed and heated at 100 °C for 15 min. The reaction was stopped on ice, and precipitated material was removed by centrifugation. A blank containing ultrapure water instead of extract was processed in parallel. Absorbance was measured at 450, 532 and 600 nm. MDA concentration was calculated as C (μmol L^−1^) = 6.45 × (A532 − A600) − 0.56 × A450. Tissue MDA content was calculated as (C × V)/W, where V is the extract volume and W is the fresh weight of the sample.

### 4.3. Electrolyte Leakage Assay

Rice shoots were excised and immersed in deionized water for 2 h. The initial conductivity of the solution was recorded as E1. Samples were then boiled for 20 min, cooled to room temperature and measured again to obtain E2. The conductivity of deionized water was recorded as E3. Relative electrolyte leakage was calculated as [(E1 − E3)/(E2 − E3)] × 100.

### 4.4. In Vitro Acetylation Assay

Plasmids encoding GPX1-His or its acetylation-site variants were transformed into *Escherichia coli* BL21 (DE3). During overnight induction, cultures were supplemented with or without 5 μM TSA and 5 mM NAM; untreated cultures served as controls. His-tagged proteins were purified, and GPX1 acetylation was analyzed by immunoblotting with an anti-acetyl-lysine antibody (PTM Biolabs, Hangzhou, China). For HDA15-dependent deacetylation assays, HDA15-GST protein and GST protein were purified from *BL21* (DE3) with pGEX-4t-1-HDA15 or pGEX-4t-1 and incubated with GPX1-His in deacetylation buffer, then the acetylation level of GPX1 were detected.

### 4.5. In Vivo Acetylation Assay

Two-week-old *GPX1*-overexpressing rice seedlings were treated with 10% PEG 6000 for the indicated times. Total proteins were extracted in RIPA buffer, and GPX1-Flag was immunoprecipitated with anti-Flag magnetic beads (Tiandi Renhe, Changzhou, China). GPX1 acetylation was detected with an anti-acetyl-lysine antibody (PTM Biolabs, Hangzhou, China), and anti-Flag immunoblotting was used to normalize GPX1 abundance. For HDA15-dependent deacetylation assays, protoplasts from 10-day-old *GPX1*-overexpressing seedlings were transfected with *HDA15-GFP* or the *GFP* control. Total proteins were extracted, GPX1-Flag was immunoprecipitated with anti-Flag beads, and acetylation was analyzed as described above.

### 4.6. Nuclear–Cytoplasmic Fractionation

Rice protoplasts transiently expressing *GPX1* or its acetylation-site mutants were collected and ground in liquid nitrogen. The resulting powder was suspended in 2 mL extraction buffer containing 20 mM Tris-HCl (pH 7.4), 25% glycerol, 20 mM KCl, 2 mM EDTA-Na_2_, 2.5 mM MgCl_2_, 250 mM sucrose, 1 mM DTT, and 1 mM PMSF [35]. The homogenized samples were passed through a 200-mesh nylon filter and centrifuged at 1500× *g* for 10 min at 4 °C. The collected supernatant was retained as the cytoplasmic fraction. The remaining crude nuclear pellet was gently washed with 3 mL NRBT buffer [20 mM Tris-HCl (pH 7.4), 25% glycerol, 2.5 mM MgCl_2_, and 0.2% Triton X-100], followed by centrifugation under the same conditions. After an additional washing step using NRB buffer [20 mM Tris-HCl (pH 7.4), 25% glycerol, and 2.5 mM MgCl_2_], the purified pellet was collected as the nuclear fraction. Rubisco and histone H3 were detected as cytoplasmic and nuclear fraction markers, respectively.

### 4.7. Subcellular Localization by Confocal Microscopy

The coding sequences of *GPX1* and its acetylation-site variants were cloned into *pAN580-GFP* vector to generate GFP fusion constructs. Protoplasts prepared from 10-day-old wild-type rice seedlings were transformed with the corresponding constructs and incubated for 12 h before fluorescence observation. During incubation, nuclei were stained with 4′,6-diamidino-2-phenylindole (DAPI). For osmotic stress treatment, transformed protoplasts were exposed to 10% (*w*/*v*) PEG 6000 for 30 min, whereas control samples were maintained in W5 solution. Fluorescence signals were visualized using a Zeiss laser-scanning confocal microscope (LSM700, Oberkochen, Gemany). GFP fluorescence was excited at 488 nm and collected at 505–530 nm, while DAPI fluorescence was excited at 405 nm and detected at 420–480 nm. Bright-field images were simultaneously acquired, and merged fluorescence images were generated to determine protein localization patterns. The representative fluorescence images were obtained from at least four independent experiments.

### 4.8. Yeast Two-Hybrid Assay

The full-length *GPX1* coding sequence was inserted into pGADT7 as the prey construct, and the full-length *HDA15* coding sequence was inserted into pGBKT7 as the bait. The two plasmids were co-transformed into Saccharomyces cerevisiae strain AH109. Transformants were selected on synthetic defined medium lacking leucine and tryptophan. Protein interaction was assessed by growth for 4 d on medium lacking leucine, tryptophan, histidine and adenine.

### 4.9. Co-Immunoprecipitation Assay

The *HDA15* coding sequence was cloned into a Myc-tagged vector and transiently expressed in protoplasts isolated from *GPX1*-overexpressing rice. After 12 h, total proteins were extracted in co-immunoprecipitation buffer containing 20 mM Tris-HCl (pH 7.5), 150 mM NaCl, 1 mM EDTA, 10% glycerol, 1 mM DTT and 1% (*v*/*v*) Triton X-100. Extracts were incubated for 3 h at 4 °C with Protein A magnetic beads pre-bound to an anti-Myc antibody. The beads were washed three times with the same buffer without Triton X-100, and bound proteins were eluted by boiling in 1× SDS sample buffer. Proteins were separated by SDS-PAGE and detected with anti-Flag (Abmart, Shanghai, China, M20008; 1:5000) and anti-Myc (Abmart, Shanghai, China, M20002; 1:5000) antibodies.

### 4.10. GST Pull-Down Assay

Purified GST-tagged protein (approximately 0.5 mg) was incubated with 20 μL Magne GST magnetic beads for 1 h at 4 °C with gentle rotation. The beads were collected on a magnetic stand and washed three times. Purified His-tagged protein (0.5 mg) was then added, and the mixture was incubated for a further 1 h at 4 °C. After three washes, bound proteins were eluted by boiling the beads in 20 μL SDS-PAGE sample buffer for 5 min. Samples were analyzed by SDS-PAGE and immunoblotting.

### 4.11. Bimolecular Fluorescence Complementation Assay

Bimolecular fluorescence complementation assays were performed with the pSPYNE and pSPYCE vectors as described previously [36]. *HDA15-YN* and *GPX1-YC*, together with the corresponding empty-vector controls, were transiently expressed in *Nicotiana benthamiana* leaves for 48 h. YFP fluorescence was recorded with a Tanon 5200 imaging system (Tanon Biomart, Shanghai, China).

### 4.12. Glutathione Peroxidase Activity Assay

GPX activity was measured from NADPH consumption at 340 nm according to previous study [13].

### 4.13. Ratiometric BiFC Assay

The full-length *GPX1* coding sequence was cloned into pDONR221-P1P4, and *bZIP68* was cloned into pDONR221-P3P2 via BP recombination (Invitrogen). A 2-in-1 LR reaction was performed using pBiFCt-2in1-CC and the two donor vectors, as previously described [23].

Agrobacterium suspensions harbouring the dual fluorescence vector *p35S:bZIP68-nEGFP-p35S:RFP-p35S:GPX1-cEGFP*, combined with *1300221-Myc* or *1300221-HDA15-Myc*, were infiltrated into *Nicotiana benthamiana* leaves. After 2 d, leaves were treated with or without 10% PEG 6000 for 30 min. Fluorescence signals were captured using a Zeiss LSM 700 confocal microscope (Oberkochen, Gemany), and the intensities of EGFP and RFP fluorescence were quantified via ImageJ software.

### 4.14. Reverse Transcription Quantitative PCR

Total RNA was extracted with TRIzol reagent (Takara, Kyoti, Japan) and reverse-transcribed into cDNA. Quantitative PCR was performed using gene-specific primers, with *OsACTIN1* as the reference gene. Relative transcript abundance was calculated using the 2^−ΔΔCt^ method. Three independent biological replicates were analyzed. Primer sequences are listed in Table S1.

### 4.15. Statistical Analysis

Statistical analyses were performed in SPSS 23.0 (SPSS Inc., Chicago, IL, USA). Multiple groups were compared by one-way analysis of variance followed by Duncan’s multiple range test. Different lowercase letters indicate significant differences at *p* < 0.05. Unless stated otherwise, data were obtained from at least three independent biological replicates.

## Data Availability

The original contributions presented in this study are included in the article/Supplementary Material. Further inquiries can be directed to the corresponding authors.

## Supplementary Materials

The following supporting information can be downloaded at https://www.mdpi.com/article/10.3390/ijms27167073/s1.

## Abbreviations

The following abbreviations are used in this manuscript:

TSA	Trichostatin A
NAM	Nicotinamide
PEG6000	Polyethylene Glycol 6000
TBA	Thiobarbituric Acid
H_2_O_2_	Hydrogen Peroxide
DTT	Dithiothreitol
TCA	Trichloroacetic Acid
MDA	Malondialdehyde
NADPH	Nicotinamide Adenine Dinucleotide Phosphate
NRBT/NRB	Nuclear washing buffer / Nuclear resuspension buffer
ROS	Reactive oxygen species
ABA	Abscisic acid
