# Supplementary material for: HDA15-Mediated Deacetylation of GPX1 Inhibits Its Nuclear Translocation and Increases Osmotic Stress Sensitivity in Rice
Source: Int J Mol Sci. 2026 Aug 7;27(16):7073. doi: 10.3390/ijms27167073 (PMC13513074; doi:10.3390/ijms27167073)
Supplement: Supplementary file 1 [file ijms-27-07073-s001.zip › Supplemental Figure.pdf]

Supporting information

HDA15-mediated deacetylation of GPX1 inhibits its nuclear translocation and increases osmotic stress sensitivity in rice

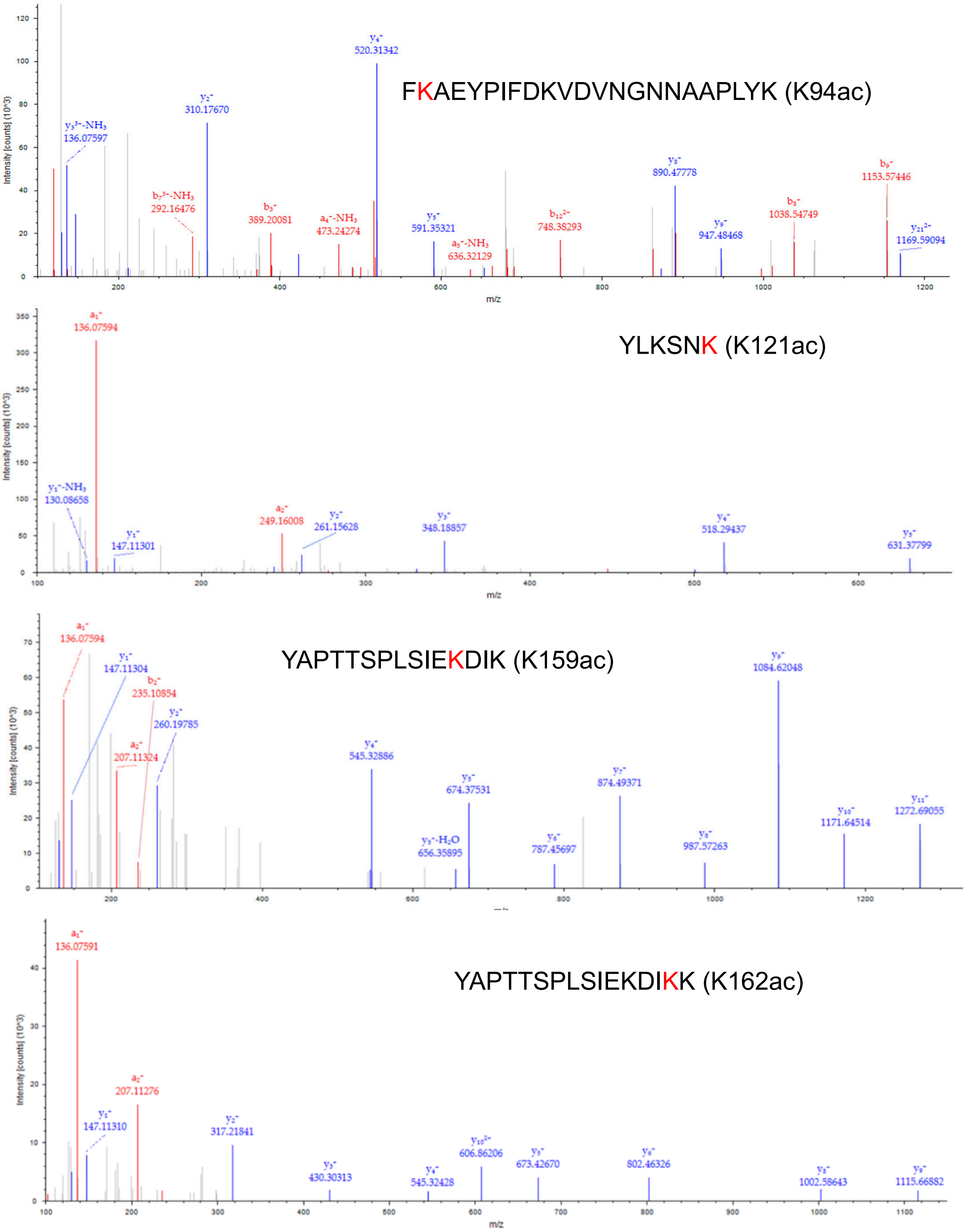

Figure S1. Analysis of acetylation sites within GPX1 using mass spectrometry. The recombinant GPX1-His protein harvested from TSA and NAM treatment were resolved by SDS-PAGE gel and gel bands corresponding to GPX1-His in were sliced. After destained the gel was subjected to in-gel digestion and analyzed by using liquid chromatography–mass spectrometry (LC-MS).

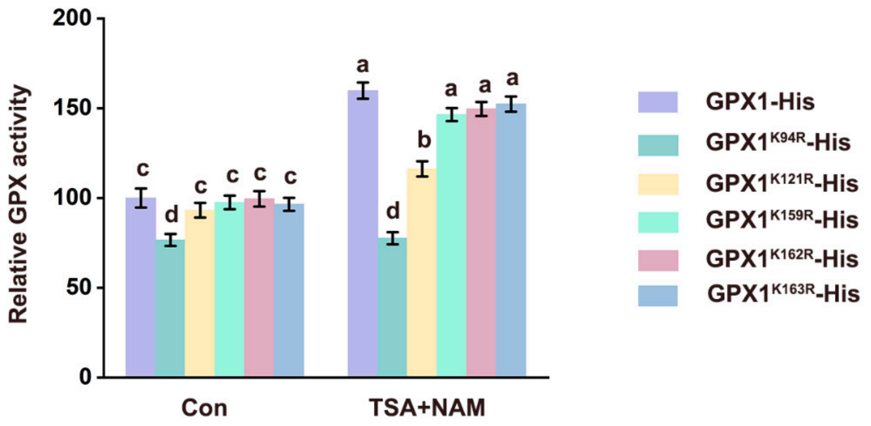

Figure S2. Acetylation affects GPX1 enzymatic activity. GPX1-His,and its lysine mutated variants harvest from the E coli treated with or without 5 μM TSA and 5 mM NAM treatment were used for GPX activity assay.

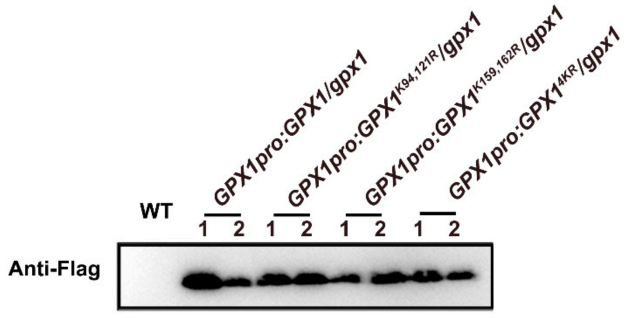

Figure S3. Characterization of different *GPX1* complementary lines.

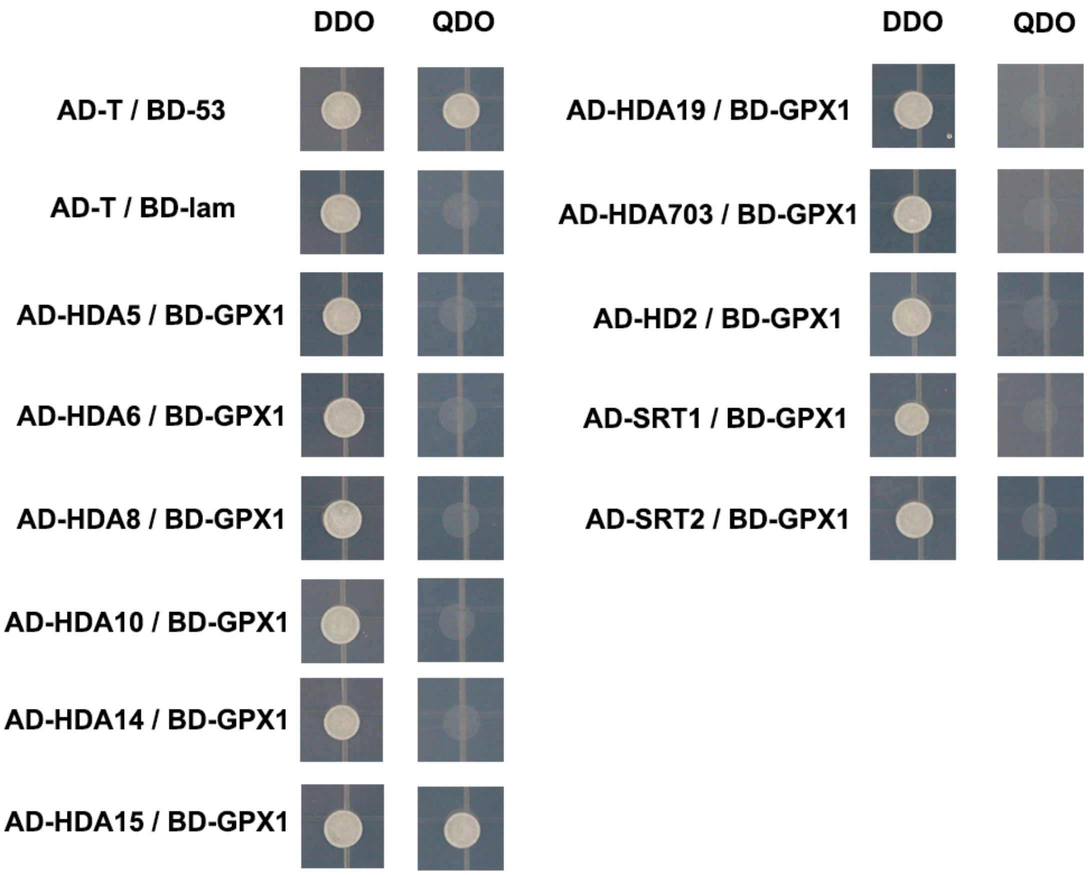

Figure S4. Verification of the potential interaction among GPX1 and other deacetylases by Y2H assay.
(A) Co-transformation of BD-GPX1 and AD-candidates of histone deacetylases in AH109. After transformation, all combination of yeasts were grown in SD-Leu/-Trp (DDO) and SD-Ade/-His/-Leu/-Trp (DDO) dropout minimal base.

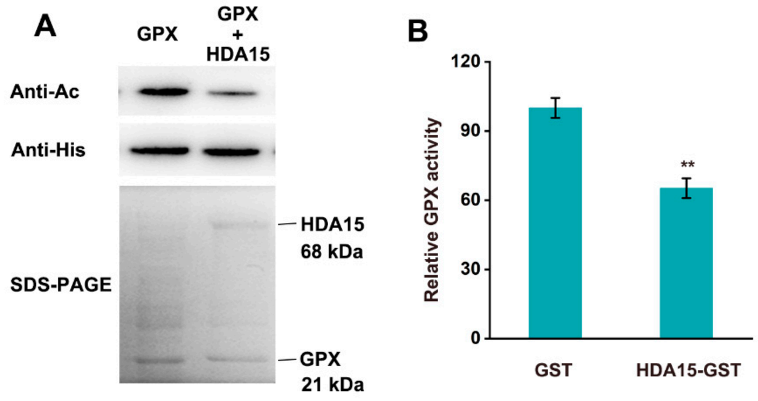

Figure S5. HDA15 deacetylates and inhibits GPX1 activity. Recombinant GPX1-His protein harvested from E coli treated with 5 μM TSA and 5 mM NAM treatment were used for in vitro deacetylation assay by GST or HDA15-GST, and then the acetylation level of GPX1 was detected by western blot assay (A), and relative GPX activity were detected (B).
